# Inflammatory Markers in Suction Blister Fluid: A Comparative Study Between Interstitial Fluid and Plasma

**DOI:** 10.3389/fimmu.2020.597632

**Published:** 2020-11-03

**Authors:** Ulrika Sjöbom, Karin Christenson, Ann Hellström, Anders K. Nilsson

**Affiliations:** ^1^ Institute of Health and Care Sciences, Sahlgrenska Academy, University of Gothenburg, Gothenburg, Sweden; ^2^ Section for Ophthalmology, Department of Clinical Neuroscience, Institute of Neuroscience and Physiology, Sahlgrenska Academy, University of Gothenburg, Gothenburg, Sweden; ^3^ Department of Oral Microbiology and Immunology, Sahlgrenska Academy, University of Gothenburg, Gothenburg, Sweden

**Keywords:** biomarkers, chemokines, cytokines, inflammation, interleukins, proteomics, proximity extension assay (PEA)

## Abstract

**Background:**

Biomarker analysis allows for the detection and prediction of disease as well as health monitoring. The use of interstitial fluid (ISF) as a matrix for biomarkers has recently gained interest. This study aimed to compare levels of inflammatory markers in ISF from suction blister fluid (SBF) and plasma.

**Methods:**

Plasma and SBF were collected from 18 healthy individuals. Samples were analyzed for 92 inflammation-related protein biomarkers by Proximity Extension Assay (PEA). Protein profiles in the two matrices were compared using traditional and multivariate statistics.

**Results:**

Out of 92 targeted proteins, 70 were successfully quantified in both plasma and SBF. Overall, plasma and SBF displayed distinct protein profiles with up to 40-fold difference in abundance of specific proteins. The levels of 25 proteins were significantly correlated between plasma and SBF and several of these were recognized as potential markers to monitor health using ISF.

**Conclusions:**

Skin ISF and plasma have unique protein profiles but many inflammatory markers are proportionally related between the matrices at the individual level. ISF is a promising biofluid for the monitoring of biomarkers in clinical studies and routine analyses.

## Introduction

Blood is the gold standard matrix for the analysis of most biomarkers in humans, i.e., for the monitoring of molecules that relate to health and for the detection or prediction of disease. Recently, interstitial fluid (ISF) has been proposed as an alternative or complementing matrix to blood for these purposes (1). ISF surrounds the cells of all tissues and provides a medium for the transport of nutrients, signals, and waste products. The capillary endothelium allows molecules to actively or passively transport between blood plasma and ISF (2). Thus, the molecular composition of plasma relates to that of ISF (3). Difficulties in harvesting ISF using non- or minimally invasive methods have limited its use in clinical and research settings. Furthermore, there is still a lack of characterized biomarkers present in ISF.

Two areas for the use of biomarkers in ISF have been proposed: I) as an alternative to blood to monitor systemic responses (4); and II) for the analysis of molecules that reflect the local environment at the site of sampling, which cannot be quantified in the systemic circulation (5). We recently showed that ISF derived from suction blister fluid (SBF) contains many lipids in similar proportions as in plasma and that ISF could be used interchangeably with plasma to monitor risk prediction biomarkers for cardiovascular disease, such as long-chain polyunsaturated fatty acids (4).

Proteomics studies have identified proteins that are unique to ISF, unique to plasma, or found in both compartments (5–7). Similarly, the transcriptome and the metabolome of ISF show resemblance to plasma but with distinct differences (8–10). These studies suggest that ISF could be used as a surrogate for plasma for the study of certain biomolecules, but also that non-overlapping information can be gained by analyzing ISF. However, most studies have focused on qualitative rather than quantitative differences between ISF and plasma.

Inflammation is characterized by the release of a large repertoire of molecules at the inflamed tissue and also into systemic circulation. Blood interleukins and cytokines are used as diagnostic biomarkers for inflammation caused by allergic reactions (11), autoimmune diseases (12), and to follow the immune system based on the response from different cell types after transplantation (13). As an example, Interleukin 6 (IL-6) is routinely used as a biomarker for the acute response of the immune system and serves as an early indicator of bacterial infection (14). Knowledge about how inflammatory markers found in blood relate to those in ISF is therefore of great clinical value for the possible future use of ISF in health monitoring.

Here, we analyzed the levels of 92 inflammatory markers in ISF from suction blisters and in plasma using semi-quantitative proteomics by Proximity Extension Assay (PEA). In comparison to mass spectrometry, which has been the primary method for analyzing the proteomes of ISF and plasma, PEA offers superior analytical sensitivity that allows the quantification of low abundant proteins, thereby capturing information on mediators with important signaling roles. Our results show that ISF and plasma have unique protein profiles but that many inflammatory markers are proportionally related between the matrices on an individual subject level.

## Materials and Methods

### Sample Collection

EDTA plasma and suction blister fluid (SBF) samples were obtained from 18 healthy human volunteers. The cohort included 13 females and 5 males with a mean age of 50.3 years (min/max 29.0/64.3 years). The same set of samples has previously been used to analyze lipidomic profiles in SBF and plasma (4). Details of the cohort (4) and procedures for the extraction of plasma and SBF have been previously described (15). All samples were collected starting between 7 a.m. and 9 a.m. after an overnight fast. Briefly, for SBF, two acrylic custom-made suction chambers (each 40 mm in diameter with three holes of 5 mm in diameter) connected to a handheld vacuum pump and tubing were placed on the volar surface of the forearm. The suction chambers were fixed with surgical tape and medical gauze and a negative pressure of 300–400 mm Hg was applied for approximately 1 h 30 min. Blisters that did not show any signs of bleeding were emptied using a sterile syringe, and the SBF was aliquoted into cryovials and stored at -80°C until analysis. Approximately 50–200 µl SBF could be collected from each individual. For blood plasma, whole blood was obtained by venipuncture and collected into 4-mL K_2_EDTA tubes (VACUETTE^®^, Greiner Bio-One GmbH, Kremsmünster, Austria). The tubes were kept at room temperature for 15 min and then centrifuged at 4°C and 2000 × *g* for 15 min. The plasma was aliquoted into cryovials and store at -80°C until analysis.

### Protein Quantification

Microneedle devices that cause minimal tissue damage and pain have been developed and are promising tools for harvesting ISF (16). These devices typically yield ISF in the nano- or microliter scale. Consequently, downstream analytical instrumentation must have low-volume requirements. We used Proximity Extension Assay (PEA) to quantify proteins relating to inflammation in plasma and SBF. The PEA technique requires only 1 µL of sample for the analysis of a panel consisting of 92 proteins and may thus be compatible with ISF volumes acquired from microneedle extractions. Although our ultimate goal is to use non-invasive methods for the collection of ISF, in this study we used SBF to establish the characteristics of this biofluid in a healthy study population, information that we hope will pave the way for future in-depth and clinical studies.

PEA protein quantification was performed by Olink Proteomics (Uppsala, Sweden) (17). The PEA technology is based on a multiplex immunoassay where each protein is targeted with a pair of antibodies that are labeled with DNA oligonucleotides. Once both antibodies bind their target antigens, the oligonucleotides can hybridize and are extended by a DNA polymerase. The DNA barcode that is formed is subsequently amplified and quantified using real-time PCR.

The lower limit of quantification (LOD) for the method was set to a value of 3SD above the mean of the background estimated from negative controls included in the assay. Values below LOD have a high risk to not be in the linear part of the calibration curve and should therefore be interpreted with caution. Proteins where less than 75% of the samples were above LOD were excluded from the analysis. The average intra-assay coefficient of variation (CV) for all analyzed proteins was 4%. 70% of the quantified proteins had intra-assay CV of <5%, 15 proteins of 5–10% CV, 2 proteins of 10–15% CV, and 5 proteins CV of >15%. Protein abundance is reported in normalized protein eXpression (NPX), an arbitrary unit in Log2 scale. A high NPX value indicates a high protein concentration although NPX values between different proteins are not comparable.

### Statistical Analyses

Multivariate data analyses were performed using SIMCA 15 (Umetrics AB, Umeå, Sweden) and univariate statistical analyses were performed using IBM SPSS Statistics, version 26 (IBM Corp, Armonk NY, USA).

Protein enrichment was tested by comparing protein NPX values between plasma and SBF using Student’s *t*-test followed by the Benjamini-Hochberg procedure to control for the false discovery rate. Linear correlations in protein abundances between plasma and SBF were measured using Pearson’s correlation coefficient.

For multivariate modeling, data were unit variance scaled. Model performances are described using R^2^X, the cumulative fraction of X variation in the model (goodness of fit in X); R^2^Y, the cumulative fraction of Y variation in the model (goodness of fit in Y); Q^2^, an estimate of the predictive ability of the model calculated by internal seven-fold cross-validation of the data.

Principal component analysis (PCA) is an unsupervised multivariate modeling method that can be used to extract overall trends and identify outliers in a dataset. In PCA, the dimensionality of the data is reduced so that it can be visualized in a few principal components where the most valuable information of the variables is retained. This allows for the identification of clustering of samples, for example, based on matrix type as in this study, and to find underlying variables that drive this separation.

Orthogonal partial least squares discriminant analysis (OPLS-DA) is a supervised modeling approach (18) aimed to identify variables that differ between two predefined groups of samples: here plasma and SBF. OPLS-DA modeling is frequently used in the context of biomarker identification. The OPLS-DA model captures between-group variation in a predictive component, and within-group variation is captured in so-called orthogonal type components. Thereby, variables that have the largest discriminatory power between sample groups can be extracted from the predictive component. As OPLS-DA models are prone to overfit data, rigorous model validation is required. OPLS-DA models were validated using cross-validation (where a high Q^2^ indicates good predictive ability), permutation test, and ANOVA of the cross-validated residuals (CV-ANOVA) (19). In the permutation test, the X variables (proteomics data) are fixed while class labels (Y, SBF, or plasma) are randomly assigned. This is repeated and each time a new OPLS-DA model is fitted using the original X and the permutated Y. R^2^Y and Q^2^Y of the original model and the permutated models can then be compared, where the latter gives a references distribution for random data. A reliable model should show greater R^2^Y and Q^2^Y than those in permutated models.

## Results

### Plasma and Suction Blister Fluid Show a Large Overlap in Detectable Inflammatory Markers

**Figure 1:**
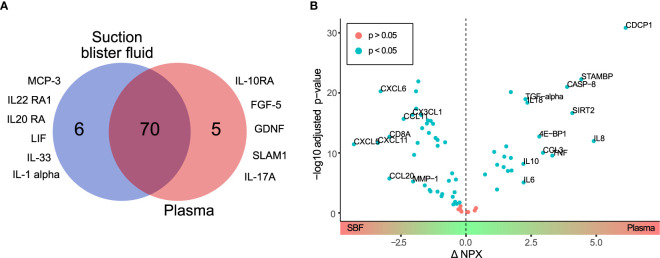
Differential protein profiles in suction blister fluid (SBF) and plasma. **(A)** Venn diagram showing proteins quantified above the LOD unique to SBF (6 proteins) and plasma (5 proteins), and common to both matrices (70 proteins). Listed are those proteins detected above the LOD only in SBF or plasma, respectively. **(B)** Volcano plot showing the difference in protein abundance (ΔNPX) between SBF and plasma (x-axis) versus statistical significance (-log10 of the p-value, y-axis). Proteins with significant differences in abundance (Student’s *t*-test, p<0.05) between SBF and plasma are highlighted in blue. Proteins showing > fourfold (ΔNPX >2) difference in abundance between SBF and plasma are labeled.

Of the 92 targeted proteins, 75 (82%) and 76 (83%) in plasma and SBF, respectively, could be quantified above LOD (**Table S1**). Only 5 proteins quantified above the LOD were unique to plasma whereas 6 of the proteins quantified above the LOD were unique to SBF (**Table S2**). Consequently, 70 proteins were successfully quantified in both plasma and SBF (**Figure 1A** and **Table S3**).

Next, fold change in protein levels between plasma and SBF was investigated. For this and further analyses, only the 70 proteins that were detectable above the LOD in both plasma and SBF were considered. Thus, proteins unique to plasma or SBF were not included in the following analyses. The difference in protein levels between the two matrices is visualized in the volcano-plot in Fig 1B. Highlighted are proteins with an NPX difference of >2 between plasma and SBF. Since NPX is in the Log2 scale, this represents proteins that show more than a fourfold abundance difference between the matrices. Of the proteins in plasma, 24 were significantly enriched, and in SBF 38 proteins were significantly enriched (**Table S3**). Thus, a considerable fraction of the quantified proteins were found in higher concentrations in SBF than in plasma.

### Plasma and Suction Blister Fluid Show Distinct Protein Profiles

**Figure 2:**
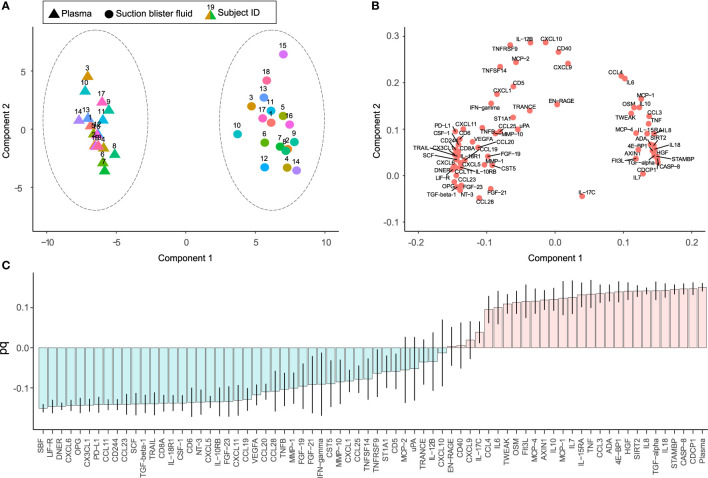
Unsupervised and supervised separation of plasma and suction blister fluid (SBF) samples based on the abundance of 70 proteins. Unsupervised principal component analysis (PCA) based on data from 36 samples collected from 18 individuals is visualized as a score plot (explained variance: Component 1 = 61%, Component 2 = 8%) **(A)**, showing clear separation of plasma and SBF samples, and corresponding loading plot **(B)** showing the correlations between underlying variables. In **(A)**, triangles indicate samples from plasma and circles from SBF, and the numbers above symbols and color indicate the subject ID. **(C)** Loading column plot of the predictive component from a supervised orthogonal partial least squares discriminant analysis (OPLS-DA), aimed to separate samples based on class, i.e., plasma or SBF. In this model, differences in protein abundance that depend on class membership are captured in one predictive component. Variables with a high magnitude pq have a large discriminatory power and contribute significantly to separate plasma and SBF samples in the model.

Protein profiles were further investigated using principal component analysis (PCA). The PCA score plot shows correlations between observations (**Figure 2A**) and the loading plot correlations between variables (**Figure 2B**) (two-component model with 61% of the variance explained by the first component and 8% by the second component, model R^2^X=0.69, and Q^2^ = 0.62). The score plot and loading plot can be compared to find relationships between observations and variables. Plasma and SBF samples were separated in the first component and in most, but not all cases the second component separated the study subjects.

In a subsequent orthogonal partial least squares discriminant analysis (OPLS-DA), samples were separated based on group classification (plasma or SBF). In this supervised model, the between-group variation is captured in one component that is orthogonal to other variations (within-group). **Figure 2C** shows the loading plot of the predictive component for the OPLS-DA model where protein abundance patterns unique to SBF are found to the left of the plot and those associated with plasma to the right. A large part of the variation between samples could be explained by matrix type according to the model (the model consisted of one predictive component, but no orthogonal components, model R^2^X = 0.61, R^2^Y = 0.98, and Q^2^ = 0.98, CV-ANOVA p = 1.7 × 10^-28^). The ability of the OPLS-DA model to discriminate plasma from SBF samples was validated by a permutation test in which the sample class was randomly permuted 999 times (**Figure S1**). There was, as expected, a large overlap between proteins showing matrix specificity in the OPLS-DA model and those showing large differences in abundance between plasma and SBF (**Figure 1B** and **Table S3**). As the primary aim of this study was to identify inflammatory markers that show similar patterns in plasma and SBF, proteins that are not predictive for the OPLS-DA model are of the greatest interest. These are primarily proteins with a pq value close to zero in the OPLS-DA loading plot (**Figure 2C**).

### Many Inflammatory Markers Show Correlation Between Plasma and Suction Blister Fluid

**Figure 3:**
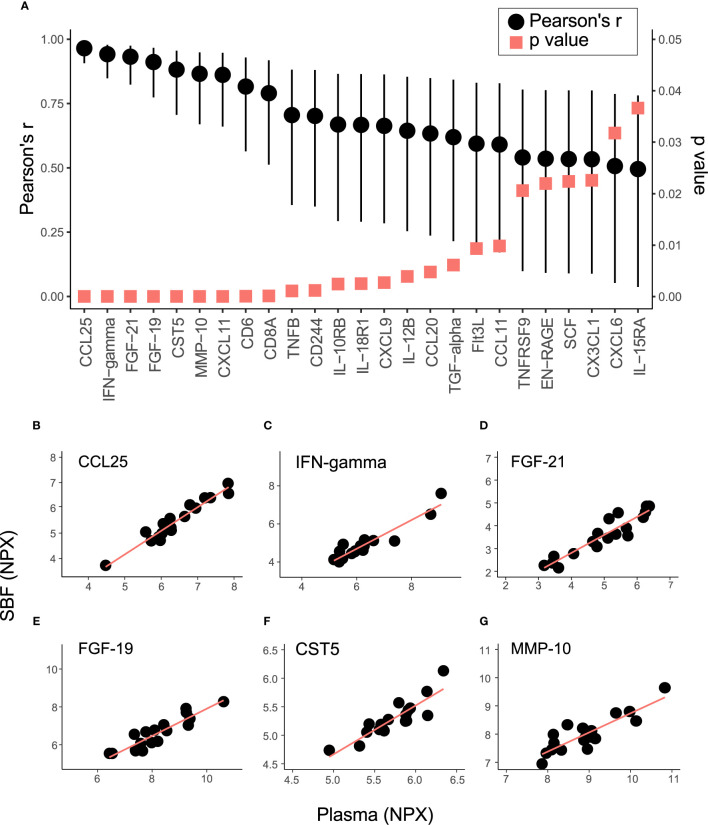
Correlation in protein abundance between SBF and plasma. **(A)** shows the Pearson’s correlation coefficient (black dots) and p-value (red squares) for the 25 proteins that demonstrated significant (p < 0.05) correlations in abundance between plasma and SBF. Whiskers indicate 95% CI. The relation between levels of Chemokine (C-C motif) ligand 25 (CCL25) **(B)**, IFN-gamma **(C)** Fibroblast growth factor 21 (FGF-21) **(D)**, FGF-19 **(E)**, Cystatin-D (CST5) **(F)**, and matrix metalloproteinase-10 (MMP-10) **(G)** in SBF and plasma are shown.

To further explore the relationship between inflammatory markers in plasma and SBF, the correlation in protein abundance between plasma and SBF was investigated. Out of the 70 proteins, 25 showed significant correlations between plasma and SBF at the p<0.05 level (**Figure 3A** and **Table S1**). In **Figures 3B–G**, abundances in plasma and SBF are shown for the six proteins that displayed the highest correlation coefficients. Not only did these proteins show high correlations between the matrices, but also the absolute abundance was largely similar between plasma and SBF.

## Discussion

Circulating biomarkers are routinely used in clinical and laboratory settings to study inflammatory responses. Here, we studied the relationship between inflammation-related markers in plasma and SBF in a cohort of healthy subjects. Our main finding is that many of the analyzed inflammatory markers show correlated abundance in plasma and SBF.

Of the 92 proteins targeted in the PEA analysis, six were quantifiable only in SBF: C-C motif chemokine ligand 7/monocyte chemotactic protein 3 (MCP-3), interleukin 20 receptor subunit alpha (IL20RA), interleukin 22 receptor subunit alpha 1 (IL22RA1), interleukin 6 family cytokine/Leukemia inhibitory factor (LIF), IL33 (interleukin 33), and interleukin 1 alpha (IL-1α). IL33 has been shown to accumulate in inflamed skin in a mouse model of poison ivy-induced allergic dermatitis and contribute to itching (20). IL-1α is present in epidermal keratinocytes and is upregulated in response to chemical exposure associated with stinging and itch (21). IL33 and IL-1α may, therefore, contribute to mediating the itch that is often perceived during the formation of suction blisters. IL33, IL-1α, and MCP-3 are reported to be secreted to blood (**Table S2**). Therefore, although enriched in SBF, these proteins are not exclusive to this compartment. IL-20RA, also found in SBF, is highly expressed in skin and is thought to be involved in epidermis function. Polymorphisms in the corresponding gene have been associated with susceptibility to psoriasis (22). Also, LIF is thought to be involved in the pathogenesis of psoriasis and the protein is released from lesional skin biopsies from psoriasis patients (23). Furthermore, mRNA transcripts and protein levels of the cytokine receptor IL-22RA1 were positively correlated with skin lesion severity in the autoimmune disease systemic sclerosis (24). Thus, most of the markers only found in SBF are associated with skin tissue and some may represent potential targets for disease monitoring.

The low proportion of proteins only detected in SBF in this study is consistent with results from Tran et al. who reported that less than 1% out of over 3000 detected proteins were unique to ISF compared with serum and plasma (7). On the other hand, Kool et al. found that 46% out of 621 detected proteins in serum and/or SBF were unique to SBF (5). In their analysis, Tran et al. extracted ISF using a microneedle array and analyzed protein abundance using liquid chromatography-mass spectrometry (LC-MS/MS) after trypsinization and fractionation of peptides. Kool et al. used SBF and depleted it from high abundant proteins, followed by trypsinization, fractionation, and detection with LC-MS/MS. Differences between studies in the methodology for extracting ISF and sample preparation may explain discrepancies in the proportion of ISF-unique proteins.

We found that a large proportion of the quantified proteins were more abundant in SBF than in plasma. This is in contrast to the overall protein concentration which is about five times higher in plasma (2, 5), but in agreement with other studies showing higher levels of inflammatory-related mediators in ISF than in serum or plasma (25, 26). CDCP1 (CUB domain-containing protein 1) was on average almost 40 times more abundant in plasma versus SBF. CDCP1 is a transmembrane protein expressed by many different cell types, including stem- and progenitor cells, and is involved in the interaction between cells and the extracellular matrix (27). CDCP1 is also abundantly expressed by the basal cells of the epidermis stratum basale, proximal to the cavity where the SBF accumulates (28). The full-length 140 kDa CDCP1 protein is proteolytically cleaved to an 80 kDa fragment by serine proteases in response to epidermal wounding (29, 30). As post-translational modifications can affect antibody binding and detection using immunochemical assays, the ability to quantify a protein may be lost after proteolysis. It is thus possible that CDCP1, as well as other studied inflammatory markers, are cleaved in response to the suction blister formation, resulting in apparently lower levels in SBF compared to plasma. CXCL5 and CXCL6 (C-X-C motif chemokine 5 and 6, respectively) were identified among significantly enriched proteins in SBF, being more than 10-fold more abundant in SBF compared to in plasma. CXCL5 and CXCL6 are involved in the chemotaxis of neutrophils through binding to the G-protein coupled C-X-C motif chemokine receptors 1 and 2 (31). CXCL5 is secreted by endothelial cells in response to infection to recruit neutrophils (32). CXCL5 (33) and CXCL6 (34) are shown to have important paracrine functions, including regulation of gene expression and signaling for cell migration and angiogenesis. Our results confirm previous studies that the proteome of ISF is distinct from that of plasma.

Levels of about one-third of the targeted proteins were significantly correlated between plasma and SBF. The six proteins that displayed the strongest correlations between matrices (depicted in **Figures 3B–G**) are discussed below. CCL25 (chemokine ligand 25) is a mucosal-associated chemokine mainly expressed in the small intestine and thymus and regulates T-cells development (35). Serum levels of CCL25 along with other chemokines and cytokines are increased in patients with inflammatory bowel disease (36). Modulation of the interaction between CCL25 and its receptor CCR9 is recognized as a therapeutic strategy against the mucosal inflammation in patients with Crohn’s disease (37), and in inflammatory bowel disease (38). The anti-inflammatory cytokine IFNγ (Interferon gamma) is an extensively studied marker used to follow T-cells reactivity after immunomodulation treatment, autoimmune-diseases (39), and T-cell malignancies (40). Among several conditions, plasma levels of IFNγ have been suggested as a clinical marker to follow disease/recovery after visceral leishmaniasis caused by *Leishmania* infection (41). FGF19 and FGF21 belong to the Fibroblast growth factor family with important functions in tissue repair and regeneration (42). FGF21 is a regulator of several metabolic pathways, including ketogenesis, gluconeogenesis, and lipolysis, and the protein is to a high degree expressed by the liver and also by skeletal muscles that releases this hormone into circulation in response to insulin stimulation. FGF21 is induced by stress and serum levels correlate with various metabolic diseases (43). FGF19 is important for cell differentiation in the brain during embryo development and acts as a negative regulator of bile acid production and transportation involved in lipid homeostasis (44). FGF19 can be used for diagnosis and potentially as a therapeutic target for bile acid diarrhoea (45) and holds promises as a marker for hepatocellular tumors (46). CST5 (Cystatin-D) was originally identified as a salivary protease inhibitor and later shown to be a potent inhibitor of human coronavirus replication (47). Recently, serum CST5 was identified as a promising early marker for traumatic brain injury (48). MMP10 is a member of the matrix metalloproteinase family and is expressed by macrophages, and has important functions in the acute response to infections in the lungs (49). Plasma levels of MMP10 are associated with albuminuria in patients with type 1 diabetes (50). In summary, we identified several proteins relating to inflammation that were well correlated between plasma and SBF. These proteins are potentials markers to follow disease progression, treatment, and recovery in ISF.

This is not the first investigation of inflammatory markers in ISF. In a rat model of inflammation, TNF showed a similar temporal abundance pattern in skin ISF and serum during endotoxemia, although the concentration was 5–10 times higher in serum (26). However, in the study, TNF levels were not detectable in serum or ISF before the endotoxemia-stimulating insult. In the same experiment, IL-1β levels increased during endotoxemia in both skin ISF and serum but were up to 50 times higher in ISF than serum. In the current study, TNF levels were on average 11 times higher in plasma compared to SBF in the cohort consisting of healthy subjects. We found no correlation of TNF levels between the two matrices. In a cohort of 44 patients with severe sepsis and 15 healthy controls, IL-10 and IL-6 levels were substantially elevated in both serum and SBF in the septic patients (25). IL-10, IL-4, and IL-6 levels correlated between serum and SBF, whereas TNF levels were unrelated. In our population of healthy individuals, levels of IL-10, IL-6, or TNF did not correlate in plasma and SBF. Levels of IL-4 in plasma and SBF were associated (Pearson’s r = 0.48, p = 0.05), however, most measurements were below the LOD. These results imply that plasma and SBF protein levels in healthy conditions and inflammation-induced conditions do not necessarily show the same intra-relationship. However, the time between the start of the blister formation and the time when the fluid is collected impact the concentration of the inflammatory markers. In the study by Koskela et al. (25), SBF was collected after 30-60 min of suction while in our study SBF was collected after approximately 1 h 30 min. SBF concentrations of the mediators TNF, IL-6, and IL-8 have been reported to be relatively low at 1 h 45 min after the initiation of blister generation but thereafter increases rapidly (15). Therefore, a local response to the blister formation can for certain markers contribute to the observed lack of correlation between plasma and SBF.

Apparent limitations of this study are that the study population was relatively small and that only healthy subjects were included. Markers identified here to show proportional relationships between plasma and SBF must be validated in patients with an ongoing inflammatory response. One of the strengths of this study is the use of the PEA technique. This technology allows for the quantification of a broad range of proteins in small sample volumes. Compared to standard proteomics approaches (e.g., mass spectrometry), PEA offers superior accuracy and sensitivity in the quantification of low abundant proteins.

In conclusion, many proteins relating to inflammation show correlated levels in plasma and SBF in healthy subjects and are potential targets for monitoring systemic conditions. Other inflammatory mediators show matrix dependent abundance profiles and may represent targets in the monitoring of local tissue-specific responses.

## Data Availability Statement

The raw data supporting the conclusions of this article will be made available by the authors, without undue reservation.

## Ethics Statement

The studies involving human participants were reviewed and approved by Regionala etikprövningsnämnden in Göteborg (no 118-16). Written informed consent for participation was not required for this study in accordance with the national legislation and the institutional requirements.

## Author Contributions

AN and AH developed the concept and design of the study. AN, US, and KC collected and prepared samples. AN and US conducted data acquisition and analysis. AN, US, KC, and AH interpreted the data and performed statistical analyses. US and AN wrote the manuscript. All authors contributed to the article and approved the submitted version.

## Funding

This work was supported by VINNOVA Swelife and Medtech4Health (2018-00245).

## Conflict of Interest

The authors declare that the research was conducted in the absence of any commercial or financial relationships that could be construed as a potential conflict of interest.
