# Supplementary figures and images for: Inflammatory Markers in Suction Blister Fluid: A Comparative Study Between Interstitial Fluid and Plasma
Source: Front Immunol. 2020 Nov 3;11:597632. doi: 10.3389/fimmu.2020.597632 (PMC7670055; doi:10.3389/fimmu.2020.597632)

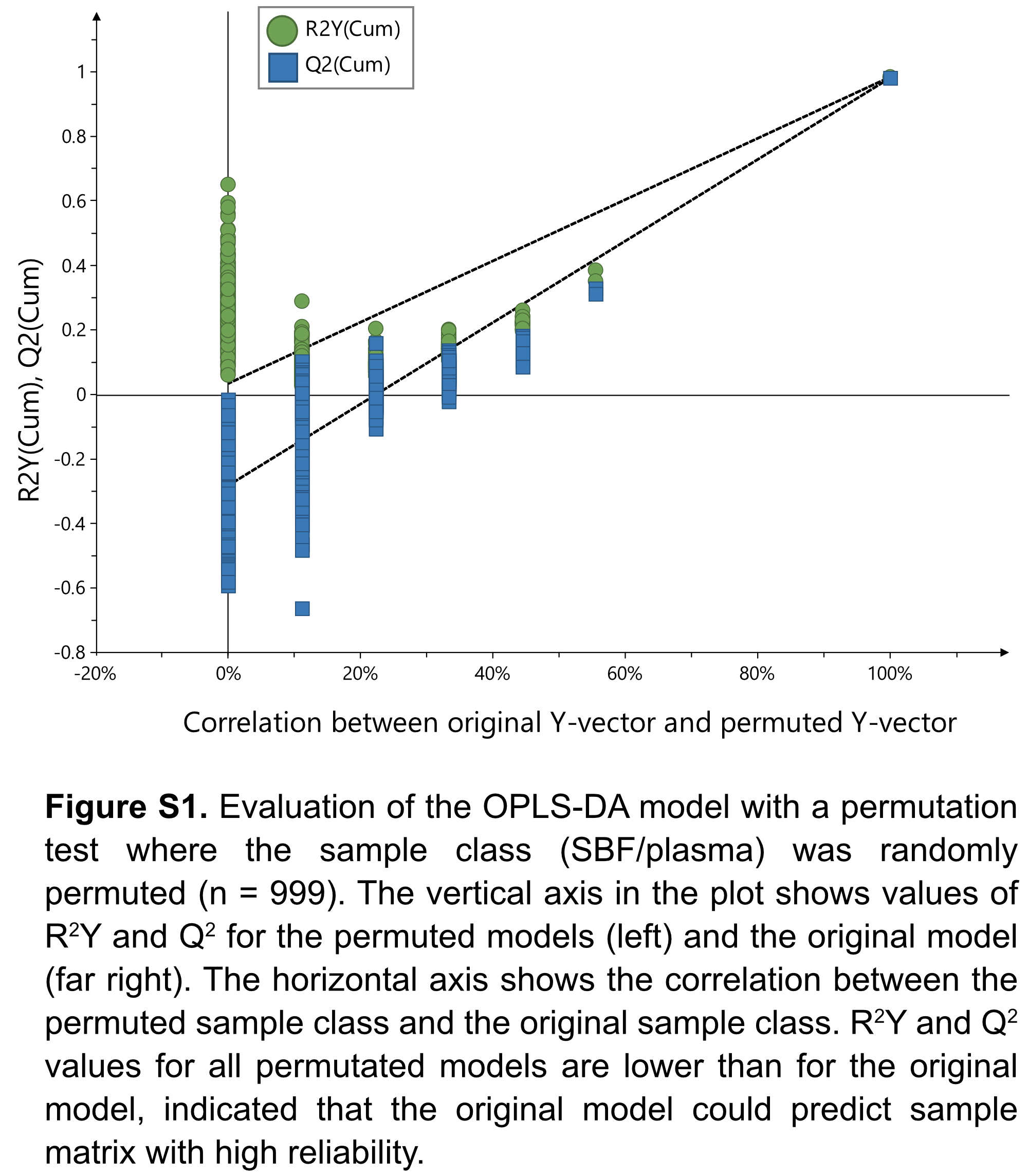

Supplement: Supplementary file 1 [file Image_1.jpeg]
